# Laparoscopic Cholecystectomy During Pregnancy: A Case Report and Review of Literature in Japan

**DOI:** 10.7759/cureus.7656

**Published:** 2020-04-12

**Authors:** Takuma Iwai, Hiroshi Makino, Tadashi Yokoyama, Masahumi Yoshioka, Hiroshi Yoshida

**Affiliations:** 1 Surgery, Nippon Medical School, Tokyo, JPN; 2 Oncology, Nippon Medical School, Tokyo, JPN

**Keywords:** cholecystectomy, pregnancy, cholecystolithiasis, laparoscopic

## Abstract

Herein we report a case of laparoscopic cholecystectomy in a 26-year-old pregnant woman with no remarkable medical history. Laparoscopic cholecystectomy was performed at 21 weeks of gestation. To prevent uterus injury, the first trocar was inserted into the right hypochondrium using the open method after marking the site of the uterus via ultrasonography. After confirming the site of the uterus, the second trocar with a balloon was inserted 3 finger widths above the umbilicus. Additional 5-mm trocars were inserted into the epigastric and hypochondrial regions. A good surgical view was obtained with a pneumoperitoneal pressure of 8 mmHg. Laparoscopic cholecystectomy was successfully performed without any complications during or after the operation. She had a normal delivery at 39 weeks of gestation. Laparoscopic cholecystectomy is a viable treatment option during pregnancy, provided there is close consultation and cooperation between obstetricians and anesthesiologists.

## Introduction

Gallstone cholecystitis is the second most common digestive disorder requiring surgery in pregnant women, after acute appendicitis [1]. Cholecystitis often recurs after initially responding to conservative treatment, and surgery is indicated if the pregnancy is adversely affected, such as if the life of the mother or the fetus is threatened [2]. It is necessary to select a minimally invasive procedure and operation time with minimal effects on the mother and the fetus. Herein we describe a case of laparoscopic cholecystectomy (LC) performed at 21 weeks of gestation, in conjunction with a review the literature on LC in pregnant women in Japan.

## Case presentation

**Figure 1:**
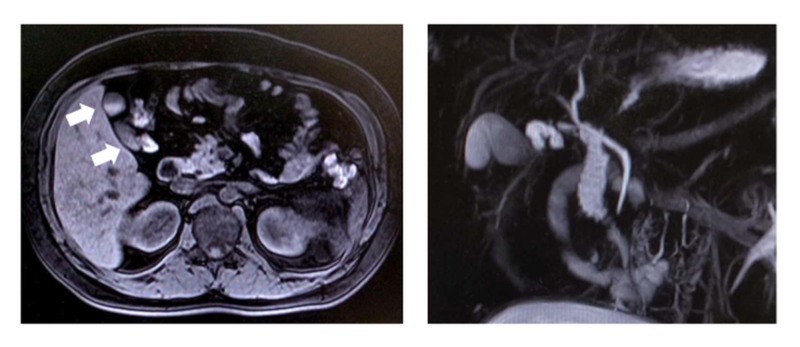
MRCP findings MRCP showed biliary sludge (arrows) in gallbladder (left) and no abnormalities in the biliary tract (right).

The patient was a 26-year-old woman (gravida 1, para 0) with no remarkable previous medical history. Right upper rib pain emerged on the 14th week of pregnancy, and ultrasonography suggested a diagnosis of cholecystitis. Her pain symptoms initially improved with conservative treatment, but the pain reemerged after the resumption of normal eating at 16 weeks of gestation, so she was referred to our department for surgery. Blood analysis revealed no abnormalities in hepatobiliary enzymes, and only mildly elevated levels of white blood cells. Her height and weight, body mass index were 164.0 cm, 76.6 kg, 27.9, respectively. She exhibited no conjunctival jaundice, conjunctival anemia, or abdominal symptoms. At the time of consultation, the gestational age had reached the 19th week and the uterus had extended to one lateral finger above the navel. Preoperative evaluation was performed with abdominal ultrasound and magnetic resonance cholangiopancreatography (MRCP) to minimize exposure to the fetus. Ultrasonography did not depict gallbladder stones or gallbladder wall thickening. MRCP depicted biliary sludge in the gallbladder, and no abnormalities in the biliary tract (Figure 1).

**Figure 2:**
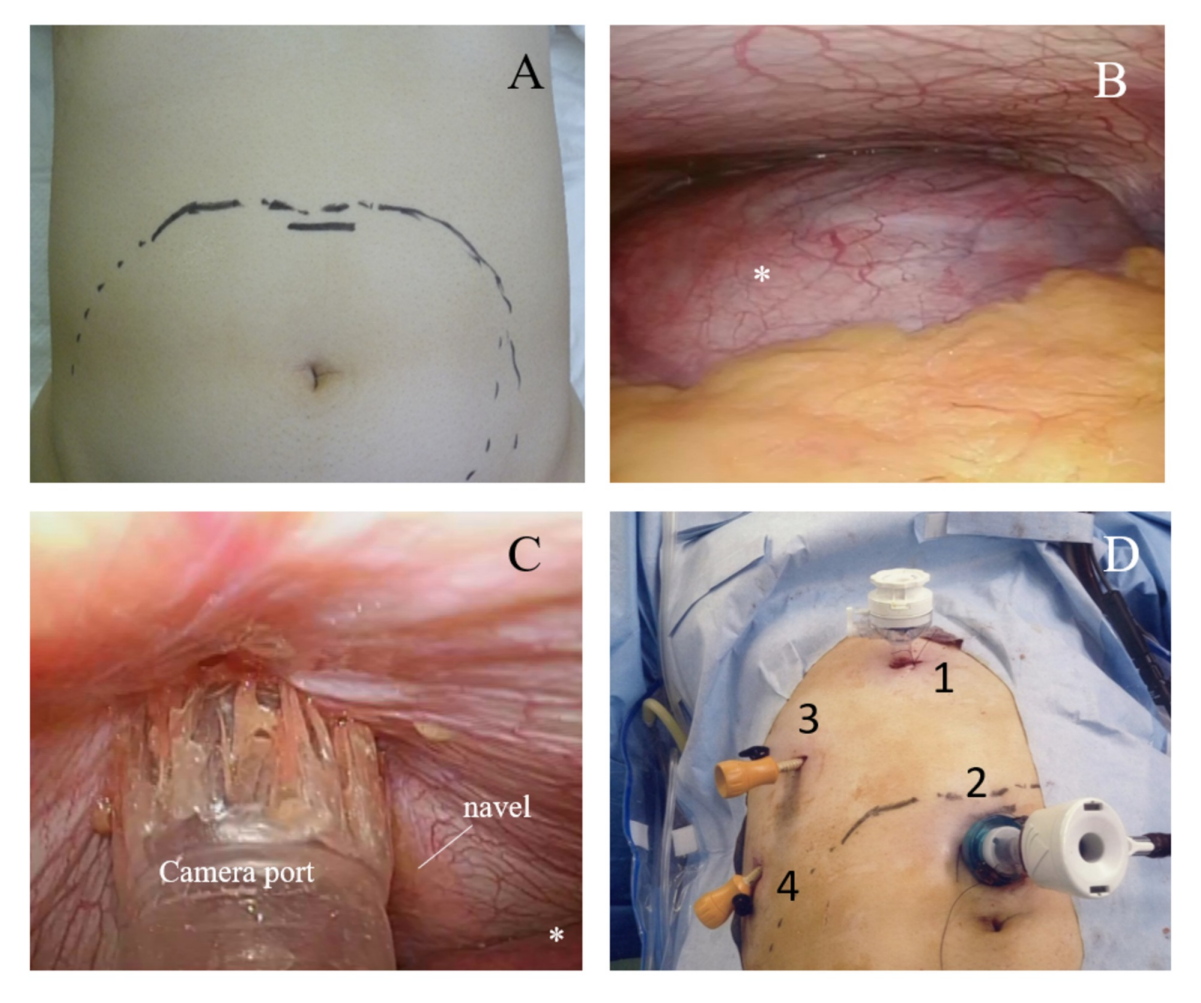
Prosedure for port insertion A) Marked the uterine fundus after the induction of anesthesia . B) The point of view from 1st port (epigastric region). The large uterine was observed (*) C) Avoiding uterine damage, the camera port was placed under laparoscopic observation. D) We performed LC with 4 ports.

Cholecystitis was diagnosed due to the biliary sludge, and the risks to the mother and fetus associated with surgical invasion and anesthesia were fully explained to the mother. After consideration of those risks, she opted for surgery. After consultation with obstetrics and anesthesiology, the operation was scheduled for 21 weeks of gestation. LC was performed under general anesthesia. An obstetrician confirmed the fetal heart rate before and after the introduction of general and epidural anesthesia. After the induction of anesthesia, the uterine fundus was confirmed via ultrasound and corresponding marks were made on the skin (Figure 2A). In order to avoid uterine damage the first port was placed from the epigastric region via the open method (Figure 2B). A camera port was then placed 3 cm cephalic from the navel (Figure 2C), and a 5-mm port was placed on the midclavicular line below the right rib and on the right abdominal anterior axillary line (Figure 2D). The body position was head‑left and supine, and the insufflation pressure was set at 8 mmHg. The transverse colon and omentum were pushed by the uterus, making it more difficult to obtain a visual field around the gallbladder neck than usual. Although there was adhesion to the surrounding tissue, the gallbladder wall was relatively soft and did not interfere with the serosa incision or detachment of the gallbladder bed. After confirming the critical view of safety, the gallbladder artery was sealed with laparosonic coagulating shears followed by ligation and separation of the gallbladder duct prior to routine LC (Figure 2).

Intraoperative cholangiography was not performed. The operation time was 93 minutes and the total blood loss was minimal. The macroscopic findings showed cholesterolosis in the mucosa of the gallbladder sporadically, but no gallstones.

A slight uterine contraction was observed after the operation, and continuous administration of ritodrine hydrochloride was initiated. The patient’s general condition was good aside from prolonged nausea the day after the operation, and her oral intake stabilized from the second day after the operation. Ritodrine hydrochloride was tapered and ended on the fourth postoperative day. She was discharged on the fifth postoperative day. The pathologically confirmed diagnosis was chronic cholecystitis with cholesterolosis. After discharge from the hospital, the courses of progression were good in both the mother and the fetus, and a normal baby weighing 2,940 g was born at 39 weeks of gestation.

## Discussion

The acute cholecystitis diagnostic criteria and severity grading system in the Tokyo Guidelines 2018 (TG18) are used globally to diagnose and rate cholecystitis [3]. Notably however, there are no clear descriptions of surgical indications or procedures to utilize in pregnant women in the TG18. To date, few cases of LC during pregnancy in Japan have been described. Those that have been reported are summarized in Table 1 [4-17].

**Table 1:** Reported cases of laparoscopic cholecystectomy during pregnancy In Japan ND= not described; *= Unknown number of days

Author	Year	Age	Pregnancy week	OP Time (min)	Intra-abdominal pressure (mmHg)	Ritodorin	Delivery week	Fetal weight (g)
Sumi et al. [4]	1996	29	15	ND	Lifting	(-)	38	3056
Morishita et al. [5]	1998	27	27	85	4	ND	39	3140
Kobayashi et al. [7]	1998	29	26	ND	8	(+) 6days	40	2400
Nakamura et al. [6]	2002	32	32	136	6	(+) *	36	1912
Sakata et al. [8]	2003	30	28	ND	8	(+) *	31	1958
Kamitani et al. [9]	2006	20	12	105	ND	(+) 5days	ND	ND
Tomono et al. [10]	2010	34	20	209	10	(+) *	39	2814
Hino et al. [11]	2010	26	19	115	Lifting	(+) 10days	29	842/626
Kudo et al. [12]	2012	40	17	115	6	(+) 3days	40	ND
Maeda et al. [13]	2013	27	19	90	8	ND	ND	ND
Kato et al. [14]	2013	37	28	170	8	(+) 14days	39	3404
Koike et al. [15]	2013	27	24	ND	8‐10	(-)	40	2824
Asaoka et al. [16]	2014	35	26	85	8	(+) 20days	36	3256
Yamamoto et al. [17]	2016	32	18	ND	8	(+) 14days	39	3620
Our case	2019	26	21	93	8	(+) 4days	39	2940

According to the Society of American Gastrointestinal and Endoscopic Surgeons (SAGES) guidelines, laparoscopic surgery can be performed at all stages of pregnancy [18]. Overseas guidelines are very helpful but should not be applied without due consideration owing to differences in the social backgrounds. We think there are three primary concerns with regard to LC during pregnancy in Japan. The first concern pertains to how LC should be applied in pregnant women, the second pertains to the optimal time to perform it, and the third pertains to the potential effects of laparoscopic surgery on the long-term prognosis of the child.

1. How should LC be applied in pregnant women?

During pregnancy, cholecystitis is a gastrointestinal disorder that requires emergency surgery second most common to appendicitis, and its incidence ranges from 0.05% to 8%. In pregnant women, supersaturation of cholesterol due to estrogen and relaxation of the smooth muscle of the gallbladder due to elevated progesterone occurs. Therefore, cholecystitis often recurs after initially responding to conservative treatment, and the risk of maternal mortality is high when gallstone pancreatitis develops [2]. In addition to this, antispasmodics for gallstone attacks promote opening of the uterine ostium via anticholinergic effects, thus limiting the administration period. Surgery is therefore considered in cases of cholecystitis recurrence. Laparoscopic surgery is advantageous in terms of postoperative pain control. Ultrasonography and MRCP have little effect on the fetus, and are recommended for preoperative evaluation during pregnancy.

2. When is the best time for LC during pregnancy?

SAGES guidelines suggest that LC can be performed at all stages of pregnancy [18]. Early pregnancy (up to 13 weeks) is a period of fetal organogenesis, and the teratogenic effects of anesthesia on a fetus at such a stage remain unclear [19]. Notably however, physical surgery space is limited during late pregnancy. Given these considerations, the guidelines of the Japanese Society of Gastroenterological Endoscopic Surgery (published only in Japanese) recommend laparoscopic surgery during the second trimester (14-27 weeks) in pregnant women. Premature births have been reported in some previous cases in which postoperative ritodrine was administered. Hence, prior consultation with a neonatal intensive care unit is required.

3. What are the effects of laparoscopic surgery on long-term prognoses in children?

Although there is one report suggesting that there are no long-term problems such as developmental disabilities associated with maternal laparoscopic surgery, the relevant evidence to date is inconclusive [20]. Indications for surgery during pregnancy should be considered carefully.

## Conclusions

LC can be performed safely in pregnant women, but the indications for it must be carefully considered and adequate preparations must be made in consultation with obstetricians and anesthesiologists.
